# The interesting relationship between APOBEC3 deoxycytidine deaminases and cancer: a long road ahead

**DOI:** 10.1098/rsob.200188

**Published:** 2020-12-09

**Authors:** Milaid Granadillo Rodríguez, Ben Flath, Linda Chelico

**Affiliations:** Department of Microbiology and Immunology, University of Saskatchewan, Saskatoon, Saskatchewan, Canada

**Keywords:** APOBEC3, somatic mutation, cancer, replication stress

## Abstract

Cancer is considered a group of diseases characterized by uncontrolled growth and spread of abnormal cells and is propelled by somatic mutations. Apolipoprotein B mRNA-editing enzyme catalytic polypeptide-like 3 (APOBEC3) family of enzymes are endogenous sources of somatic mutations found in multiple human cancers. While these enzymes normally act as an intrinsic immune defence against viruses, they can also catalyse ‘off-target’ cytidine deamination in genomic single-stranded DNA intermediates. The deamination of cytosine forms uracil, which is promutagenic in DNA. Key factors to trigger the APOBEC ‘off-target’ activity are overexpression in a non-normal cell type, nuclear localization and replication stress. The resulting uracil-induced mutations contribute to genomic variation, which may result in neutral, beneficial or harmful consequences for the cancer. This review summarizes the functional and biochemical basis of the APOBEC3 enzyme activity and highlights their relationship with the most well-studied cancers in this particular context such as breast, lung, bladder, and human papillomavirus-associated cancers. We focus on APOBEC3A, APOBEC3B and APOBEC3H haplotype I because they are the leading candidates as sources of somatic mutations in these and other cancers. Also, we discuss the prognostic value of the APOBEC3 expression in drug resistance and response to therapies.

## 1. Introduction

Cancer is a generic term used to describe more than 100 diseases in which cells grow out of control in any part of the body. It has long been known that cancer has a basis in somatic mutations that alter a diversity of cellular functions resulting in sustained proliferative signalling, evasion of growth suppressors and genome instability [1]. Mutations contribute to genomic variation, and may result in neutral, beneficial or harmful consequences for an organism. Cancer genomic sequencing studies have identified mutational signatures that reflect the corresponding causes of these mutations. Mutagenesis originates from exogenous sources and endogenous sources that reside intracellularly [2–5]. Exogenous sources include radiation and chemical damage [2,6,7] and endogenous sources are DNA replication errors, the inability to repair the DNA damage after it has been triggered, and agents that impair DNA directly [8].

The first evidence revealing the apolipoprotein B mRNA-editing enzyme catalytic polypeptide-like 3 (APOBEC3) family of enzymes as endogenous sources of somatic mutations found in human cancer were provided in 2012 by Nik-Zainal *et al*. [9] and Roberts *et al*. [10]. Then, in 2013, the extensive resources generated by The Cancer Genome Atlas (TCGA) revealed APOBEC mutagenesis in multiple cancer types [11–13]. There are seven APOBEC3 (A3) enzymes in humans (A3A-H, excluding E) that are capable of inducing DNA mutations through the deamination of cytosine to form promutagenic uracil on single-stranded (ss) DNA. This is the main mechanism by which these enzymes restrict replication of endogenous retroelements, DNA viruses and RNA viruses [14–18]. Some of the A3 enzymes can also deaminate RNA, although the physiological function is yet to be determined [19–22].

The studies of A3 enzymes have been predominantly focused on understanding their antiviral activities, biochemical properties or retroactive analysis of mutated human genomic DNA sequences, but still little is known about the step-by-step process of how these enzymes have a propensity to act in an ‘off-target’ fashion in human genomes. This new concept in cancer involves the overexpression of these enzymes in a ‘wrong cell’ or at the ‘wrong time’ and catalysing the ‘off-target’ deaminations in human ssDNA intermediates, with implications in somatic mutagenesis. Those somatic mutations are found in approximately 15% of sequenced human tumours [3,5,11,12,23] with A3A, A3B and A3H haplotype I (Hap I) as leading candidates [3–5,13,24].

This review addresses the interesting relationship between A3 enzymes and cancer. There are several excellent reviews about this topic [25–31]; however, in this review, we bring together for the first time the clinical, molecular, genetic and biochemical perspectives regarding A3A, A3B and A3H Hap I for four main cancers where there is the most published information (breast (BRCA), lung, bladder (BLCA) and human papillomavirus (HPV)-associated cancers), although A3-induced mutations are involved in at least 16 cancer types [3,5,11,12,24,32–38]. This review brings ideas together from multiple disciplines and enables novel conclusions, hypotheses and gaps of knowledge in the field to be identified. First, we explain the physiological functions of these enzymes and the biochemical basis of the somatic mutagenesis to provide a better understanding on how these enzymes are involved in cancer. Second, using clinical, molecular and genetic information, this review discusses the relevance of these enzymes for the prognosis or treatment of cancer.

## 2. A3 physiological functions

A3 enzymes belong to a larger cytidine deaminase family, which in humans also includes activation-induced cytidine deaminase (AID), APOBEC1, APOBEC2 and APOBEC4 [15]. The human A3 family was identified in 2002 and is composed of seven members (A3A, A3B, A3C, A3D, A3F, A3G and A3H) that are encoded by genes located on chromosome 22 [39]. The expression of A3 enzymes is tissue and cell type-specific [40,41] and they are expressed at different levels in immune cell populations such as CD4^+^ (naive and memory subsets) and myeloid cells. Peripheral blood leucocytes express transcripts for all A3 enzymes, with A3A and A3G being the most represented [40]. The expression of A3A is specific to cells of the myeloid lineage, whereas A3G is highly expressed in CD4^+^ T lymphocytes [40,41]. The A3 enzymes are also present in non-immune tissues (e.g. epithelial, lung, ovary and adipose tissue), sometimes constitutively or after upregulation due to viral infection [40,42–44].

The A3 enzymes deaminate cytosine in ssDNA which forms uracil, which is not a natural base in DNA and is treated as a promutagenic lesion [45,46]. The intrinsic deaminase activity of these enzymes is mitigated by DNA repair processes, which usually restores the original DNA sequence in an error-free manner through base excision repair (BER) that can remove this lesion from DNA through the action of uracil DNA glycosylase (UNG) [45,46]. However, A3-mediated deamination of cytosines to uracils can also lead to C-to-T mutations directly through DNA replication using uracil as a template or other mutations by translesion synthesis (TLS) polymerases that insert incorrect bases opposite abasic sites after uracil removal. According to yeast experiments, the observed C-to-G transversions that are linked to A3 deamination activity may be caused by TLS bypass over an abasic site by REV1 and DNA polymerase *ζ* after uracil base removal by UNG2 [47,48]. The yeast studies also observed that a smaller number of C-to-A transversions occurred as a consequence of the generation of abasic sites after removal of uracils, but by a still unclear mechanism [48]. The loss of Rev1 catalytic activity does not have any apparent effect on the ability to insert A opposite abasic sites, indicating that polymerase activities besides that of Rev1 are entirely responsible for A insertion events [49].

The subcellular localization of A3 enzymes is important for their biological functions. A3 enzymes can have different subcellular localizations: A3G, A3F and A3D are cytoplasmic, whereas A3A, A3C and A3H display pan-cellular localization and A3B is localized predominantly in the nucleus [50]. The localization differences (cytoplasmic or nuclear) place these enzymes in the best position to inhibit different viral pathogens, e.g. human immunodeficiency virus (HIV)-1 (cytoplasm) or retroelements (nucleus) and the activities of A3s in the same cellular compartment are redundant [51–53]. Primarily, the A3 enzymes constitute an innate barrier to retroviruses including HIV-1, endogenous retroelements, DNA viruses (e.g. hepatitis B virus (HBV), adeno-associated virus, herpes simplex virus 1, HPV, Epstein–Barr virus (EBV)) and RNA viruses such as human coronavirus [14–18].

The restriction of the replication of these viruses occurs primarily through the deaminase-dependent activity of A3 enzymes which results in hypermutated and inactivated viral genomes. A3G was the first A3 enzyme demonstrated to have restriction activity against HIV infection through G-to-A mutations in the sense DNA strand creating non-infectious virions when uracils in the anti-sense DNA were used as a template in DNA synthesis. Also, uracil containing viral DNA can be degraded by host DNA repair enzymes [54,55]. Deamination-independent antiretroviral mechanisms for A3G have also been described, such as inhibition of HIV reverse transcriptase [56]. Similar to A3G, A3F and A3H can function as HIV restriction factors through encapsidation into budding virions to exert their antiviral activity in newly infected cells, whereas A3A can restrict infection directly in the target cells where it is endogenously expressed [57]. However, this only occurs if A3 enzymes bypass the HIV viral infectivity factor (Vif), which facilitates their ubiquitination and degradation [58,59]. Initially, it was thought that this intricate interplay of virus and host interactions was specific to HIV and A3s. However, it has been shown that even for DNA viruses, such as EBV, there exists a viral protein, in this case BORF2, that binds to and inhibits A3B [60]. Otherwise, A3B would suppress viral replication through deamination of cytosines. There may be other viruses that express an A3 antagonist protein, but currently, other viruses that A3s can restrict, such as coronavirus [18] and HBV [61], are not known to have a counteraction mechanism. In addition to the role of A3 enzymes in intrinsic antiviral responses, these enzymes are also involved in innate and adaptive immunity. It was documented that A3G has a role in the recognition of HIV-infected cells by NK cells and CD8^+^ cytotoxic T lymphocytes [62,63], whereas A3A is able to edit the transcripts of several genes associated with viral pathogenesis, in monocytes and macrophages [19].

Another important physiological function of A3 enzymes is the restriction of endogenous retroelements. These retroelements including those containing long terminal repeats (LTR) like endogenous retroviruses, as well as non-LTR elements like long interspersed nuclear elements (LINEs) and short interspersed nuclear elements (SINEs), could have provided the evolutionary pressure necessary for the maintained expansion of the A3 locus in primates. Non-LTR elements, including LINE1 and Alu, are restricted by human A3 enzymes and this mechanism appears to be deamination-independent, except for A3A, in contrast with the restriction mechanism for LTR containing endogenous retroviruses [64–68]. Endogenous retroelements and their mobility are believed to have played a central role early in shaping the human genome during speciation [66,69]. Ultimately, cells have devised strategies to defend and preserve genomic integrity and the evolution of the A3 family has likely played a prominent role in this defence and in diversifying the retroelements to make them more useful for the host species [66].

At a population level, there is a cost to this good defence. When the enzymes localize to the nucleus and have access to the genomic DNA, there is the risk of ‘off-target’ activity against host genomic DNA and the potential for mutagenesis through error-prone DNA repair systems. In general, these mutations occur randomly across the genome during our lifetime and sometimes the ‘wrong combination’ of somatic mutations can transform a normal cell into a tumoural cell.

## 3. Biochemical basis of A3 enzyme activity

**Figure 1.**
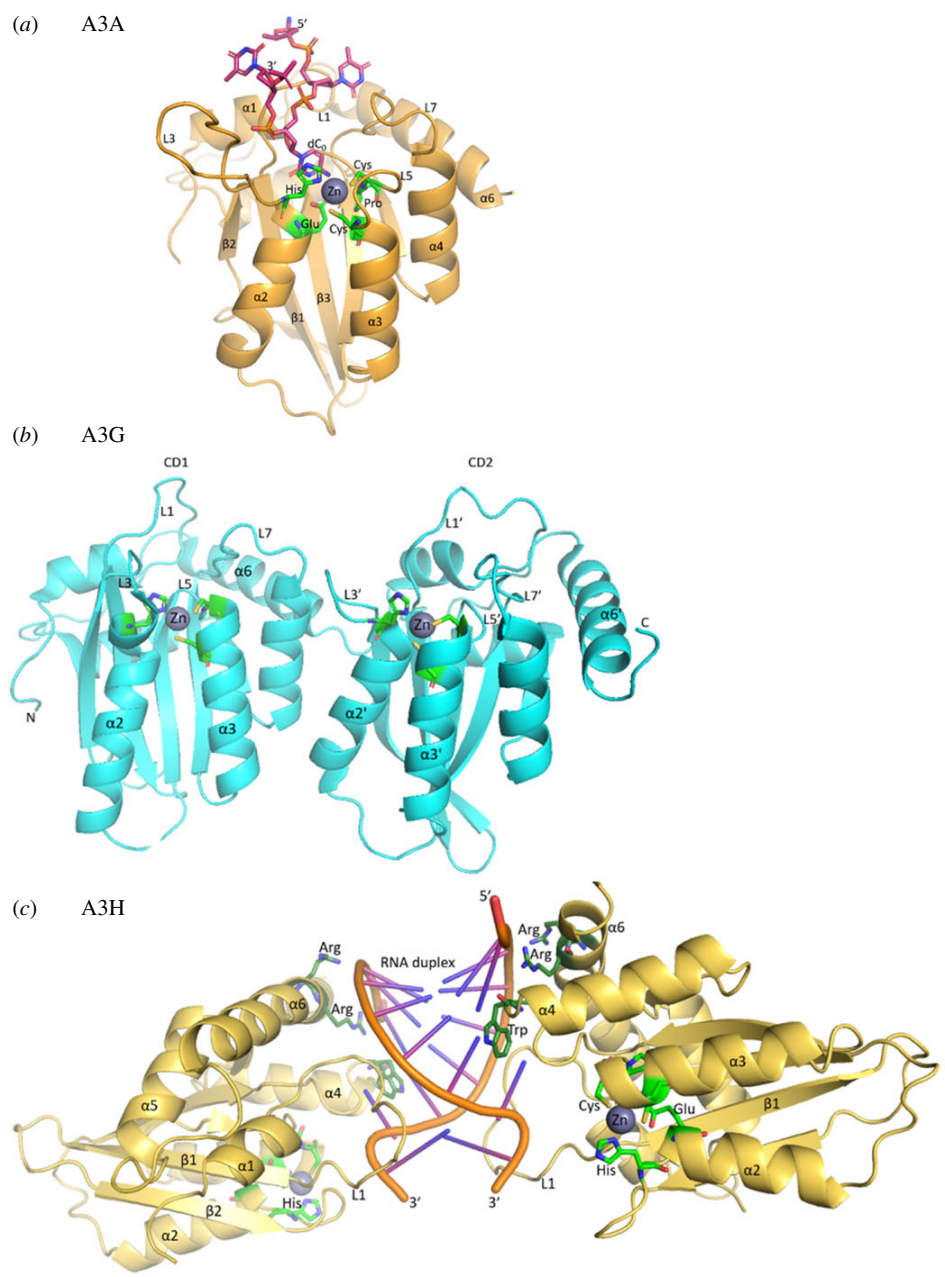
A3 structures. (*a*) The core domain and active site of A3s, showing the single-domain A3A as an example. PDB 5KEG shown with modification to include the catalytic glutamate on α-helix 2 (α2) instead of alanine [70]. The consensus sequence His-*X*-Glu-*X*_23–28_-Pro-Cys-*X*_2–4_-Cys is represented as green residues coordinating a Zn atom. Pink carbon atoms represent the four nucleotide DNA (5′- dT2, dT1, dC0, dT-1 -3′) substrate. Blue, nitrogen; red, oxygen; yellow, sulfur and orange, phosphorus. (*b*) The double-domain A3G (PDB: 6P40) with loop 7' (L7') being the principal determinant of sequence specificity in the C-terminal domain (CD2) and loop 7 (L7) being a principle determinant for processivity and oligomerization (not shown) in the N-terminal domain (CD1) [71]. In both the CD1 and CD2, loops 1 (L1, L1'), 3 (L3, L3') and 5 (L5, L5') also contribute to DNA binding. (*c*) A3H RNA-mediated dimer (PDB 5W3 V) [72]. The catalytic residues (light green) coordinate the zinc atom. Key residues lying on α-helix 6 (α6) and loop 7 (L7) electrostatically mediate the protein DNA interface (dark green). The highlighted Trp residue lies on L7 forming a critical stacking interaction with nucleotide 3 of the RNA duplex.

Four members of A3 enzymes contain two zinc-coordinating domains (A3B, A3D, A3F and A3G) and three members contain one zinc-coordinating domain (A3A, A3C and A3H) with the consensus sequence His-*X*-Glu-*X*_23–28_-Pro-Cys-*X*_2–4_-Cys (figure 1*a*) [73,74]. For A3 enzymes with two Z-domains, only the C-terminal domain is catalytically active, although both domains coordinate zinc (figure 1*b*) [75–78]. Among all these enzymes, A3A has the highest catalytic activity [79]. The APOBEC enzymes induce mutations in a sequence-specific manner and the majority of A3 family members preferentially deaminate the central cytidine in 5′HTCW trinucleotide motifs (where H = A, C or T and W = A or T; the deaminated based is underlined) within ssDNA substrate, except A3G (5'CCCA) that deaminates cytidines in a different sequence motif [80].

The A3 enzymes can bind cellular RNA and this can have an impact on their enzymatic activity. A3D, A3G and A3F all bind cellular RNA and form a ribonucleoprotein high molecular weight molecule that is catalytically inactive *in vitro* unless treated with RNaseA [81–83]. Some studies have suggested that RNA binding could be a mechanism to inhibit A3 activity on human genomic DNA and promote cytoplasmic localization [84,85]. In the case of A3H, there is a clear association of RNA binding and cytoplasmic localization, suggesting that RNA is involved as a regulatory mechanism. A3H uses RNA in cells to dimerize (figure 1*c*), which promotes enzyme activity [86]. A3H has seven major haplotypes (Hap I–VII) with A3H Hap II mostly localized to the cytoplasm and A3H Hap I mainly localized to the nucleus [87], and only A3H Hap I is involved in somatic mutagenesis [5]. By contrast, A3B is strongly inhibited by RNA indicating that A3B likely requires activation for activity in cells [24], similar to what was reported for AID [88,89] and A3A does not bind cellular RNA [24,83]. A3H Hap I has minimal inhibition by RNA, consistent with primarily but not exclusively nuclear localization, suggesting an intermediate phenotype between A3B and A3A [84,86,87]. The negative regulatory role of RNA in suppressing the DNA deaminase activity is likely to be relevant to preventing the accumulation of somatic mutations in development, ageing and cancer [84].

## 4. Biochemical basis of A3-induced somatic mutagenesis

**Figure 2.**
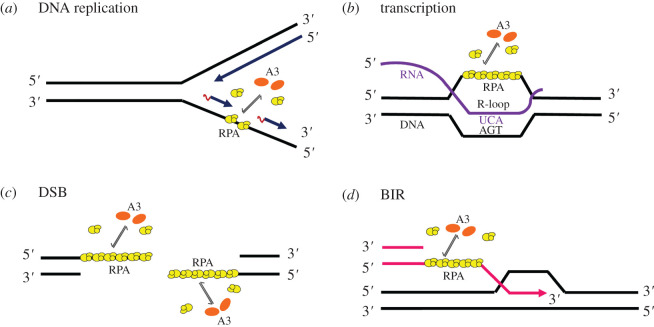
A3 deamination of cellular ssDNA templates. Single-stranded substrates of A3 deamination include ssDNA intermediates generated at replication forks (*a*), during transcription (*b*), at DSBs (*c*) and those generated during BIR (*d*). RPA (yellow) is bound to ssDNA and for A3 enzymes (orange) to access the ssDNA, they must be able to compete with RPA. The ssDNA mutagenesis during transcription can take place only if the lesions in the non-transcribed strand persist until DNA replication, then they can be fixed into mutations by TLS. By contrast, resolution of the R-loops provides the undamaged template for excision repair and prevents mutagenesis. For the mutagenesis associated with both DSB and BIR, there is no template for accurate excision repair. An A3 monomer is shown, although A3B forms larger oligomers and A3H is a dimer. Only A3A is monomeric.

The A3 enzymes that induce somatic mutagenesis must not only be able to localize to the nucleus but must also be able to deaminate transiently available ssDNA created during dynamic processes such as transcription, replication or double-strand break (DSB) repair. These enzymes can cause mutations in the cellular genome at replication forks (figure 2*a*) or within transcription bubbles (figure 2*b*), depending on both the physiological state of the cell and the phase of the cell cycle during which they are expressed [44]. The deoxycytidine deaminase activity of A3A, A3B and A3H Hap I, as leading candidates, has been implicated in cancer and tumour evolution by providing the cells with a diverse pool of mutations [25,90]. Previous experiments of the capability of the A3s to affect cell cycle progression suggest that also A3D might play a role in genomic mutation [91]. Interestingly, DNA damage can cause cytoplasmic A3G to enter the nucleus, but evidence suggests that A3G cytidine deamination promotes DNA repair [92]. Previous studies have shown that A3 enzymes primarily deaminate genomic DNA during replication and favour the lagging strand due to the greater abundance of ssDNA from discontinuous synthesis [93–97]. Single-stranded substrates of A3 deamination include single-stranded intermediates at replication forks (figure 2*a*), DSBs (figure 2*c*) and those generated during break-induced replication (BIR) (figure 2*d*) [98]. Phosphorylation of the Ser-139 residue of the histone variant H2AX, forming *γ*H2AX, is an early cellular response to the induction of DSBs. Detection of this phosphorylation event has emerged as a highly specific and sensitive molecular marker for monitoring DNA damage initiation and resolution. The potential for A3 enzymes to cause DSBs has been discussed due to increased *γ*H2AX in response to ectopic A3 expression in cell lines [3,99]. The replication stress induced by hydroxyurea treatment of cancer cells expressing A3A led to increased *γ*H2AX [100] and A3B expression also caused induction of a DNA damage response characterized by *γ*H2AX and ssDNA-binding protein (RPA) phosphorylation [101]. A model was suggested in which BIR was observed to provide a substrate for clustered mutations during DNA replication [102,103]. During BIR, only one end of a DSB can be repaired and this uncouples the replication bubble so that the lagging strand is delayed behind the leading strand and ssDNA accumulates, providing a substrate for clustered mutations.

For enzymes that modify the DNA, processivity has always been thought of as essential for enabling deamination of multiple cytidines in a single enzyme–substrate encounter. The loop 7 and/or helix 6 structures have been shown for all A3 enzymes to be important for processivity on and binding to ssDNA, with loop 7 being the principal determinant of sequence specificity, but loops 1, 3 and 5 also contributing to DNA binding in both single domain (e.g. A3A; figure 1*a*) and double domain (e.g. A3G; figure 1*b*) A3s [104–112]. The enzymes that do not use an energy source to move on DNA, such as APOBECs, can search for their DNA-specific target motif using a mechanism called ‘facilitated diffusion’ [113,114]. This mechanism includes sliding along DNA, microscopic dissociation–reassociation events between closely spaced sites (jumping or hopping) and intersegment transfer [113]. The ‘facilitated diffusion’ enables deamination of multiple closely spaced targets on DNA [115,116], but if an enzyme undergoes macroscopic dissociation from DNA after one turnover, it is considered to be non-processive or distributive. The processivity of several members of the AID/APOBEC family members on ssDNA has been reported and the results have implications for the finding that mutations associated with AID or A3s are often clustered [117,118].

Adolph *et al*. in 2017 [119] reported the first biochemical characterization of A3B and compared properties of A3B, A3A and A3H Hap I on substrates relevant to catalysing cytidine deaminations in genomic DNA. They found, using *in vitro* transcription and replication model systems that surprisingly, A3A, a non-processive enzyme, was equally effective in deamination during replication as A3B and A3H Hap I that were processive enzymes. A3B and A3H Hap I cycled between ssDNA substrates, but also maintained processivity while bound to a substrate, whereas A3A simply cycled on and off substrates rapidly. The ability to cycle between ssDNA substrates was important to not only quickly access the transiently ssDNA being replicated but also ssDNA bound by RPA, suggesting that the ability to compete for ssDNA rather than processivity was most important for A3s to induce ‘off-target’ deaminations. In order to access the protected ssDNA, A3 enzymes would also have to displace RPA by a mechanism known as ‘facilitated dissociation’ [120,121], similar to how excess RPA or RAD51 was previously found to be able to exchange with bound RPA on the ssDNA. The *in vitro* study of A3 enzymes competing with RPA-saturated ssDNA for DNA binding showed that rapid cycling was required for RPA facilitated dissociation by A3A, A3B and A3H Hap I. By contrast, A3G, which is one of the most efficient enzymes for HIV-1 restriction, has a decreased frequency of cycling between ssDNA substrates and was greatly inhibited in the presence of RPA with a 10-fold decrease in specific activity, in contrast with A3A, A3B and A3H Hap I that had at most a twofold decrease in specific activity [119]. These results demonstrate that during replication stress where larger amounts of ssDNA accumulate, the protective RPA barrier is less effective against A3A, A3B and A3H Hap I.

The inherent properties of the A3s also determine if the enzymes deaminate the ssDNA during replication or transcription, with transcription being more selective [119]. Although A3A, A3B and A3H Hap I could deaminate during DNA replication or on ssDNA bound by RPA, the amount of deamination correlated with the ability of the enzymes to cycle between ssDNA substrates [119]. The results obtained using *in vitro* transcription and replication model systems were in good agreement with previous studies done in yeast. For A3A, there was an association of mutations in a yeast system with the non-transcribed strand, but the majority of mutations correlated with the lagging strand of replication [96]. In the case of A3B, the mutations correlated with the lagging strand of replication, not the ssDNA on the non-transcribed strand generated during transcription [96], probably due to A3B oligomerization exceeding the size limitation of the transcription bubble [119,122]. On the basis of the available data, A3B would be unable to deaminate ssDNA generated during transcription, unless stable R-loops were to form [119]. The ssDNA mutagenesis during transcription can take place if the lesions in the non-transcribed strand persist until DNA replication, at which time they can be fixed into mutations by TLS and this would result in a mutation bias favouring the non-transcribed strand [98]. No cellular studies examining A3H Hap I in this regard are available, but a study using a bioinformatics approach has suggested that A3H Hap I could act early in lung cancer mutations and possibly contribute to the APOBEC signature in A3B-null BRCA [5]. This is in contrast with A3B-induced mutations that were absent early in tumour formation but then suddenly arose and were maintained at a high level. One limitation of the precedent studies in which transcription has not been observed as a dominant form of ssDNA substrate was that the end-point analysis from tumours or after months of expression of A3 enzymes in yeast takes into account only accumulated but not temporal mutations [93–97]. Whether A3s enzymes play a role in driving carcinogenesis and/or tumour progression needs further specific studies in each cancer type. Determining how and when (during replication, transcription or repair) A3 enzymes can deaminate genomic ssDNA will allow us to identify if they cause cell transformation, contribute to a mutator phenotype or both.

## 5. A3 enzymes and cancer

Cancer is considered a group of diseases that involve dynamic changes in the genome. Genomic instability is one of the hallmarks of cancer that cause both aberrant chromosomal architecture and mutational changes at the single nucleotide level [1]. The diversity created by the above-mentioned processes provides the substrate for selection within tumours. Although an elevated mutation rate is not necessarily a requirement for the initiation of a tumour, it will likely contribute faster to tumour evolution and adaptation [123]. In addition to other endogenous mutational factors, now it is well known that some members of the A3 enzymes are an endogenous source of somatic mutations found in approximately 15% of sequenced human tumours such as, BRCA, bladder, cervix, lung (adenocarcinoma and squamous cell carcinoma), head and neck, myeloma, renal cell carcinoma, stomach and thyroid [3,5,11,12,24,32–38]. All cancers are caused by somatic mutations that are the aggregate outcome of one or more mutational processes operative through the life of the cancer patient [9,124]. Different mutational processes often generate different combinations of mutation types, termed ‘signatures’. Each mutational process leaves a characteristic mutational signature determined by the mechanisms of DNA damage and repair. The APOBEC family of cytidine deaminases generates particular genome-wide mutational signatures and a signature of localized hypermutation called ‘kataegis’ or ‘mutation clusters’ [9,10,125,126]. Two signatures characterized by C-to-T and/or C-to-G mutations at TpCpX trinucleotides were identified (the underlined base is the mutated base and X can be any base) in several cancer types and are among the most common mutational signatures found in human cancer [9,11]. These signatures have been designated Signatures 2 and 13 [11]. Signature 2 is composed predominantly of C-to-T transitions with fewer C-to-G transversions and Signature 13 is dominated by C-to-G transversions at a TpCpX sequence context and due to error-prone repair of APOBEC-induced uracils [9,11].

APOBEC deoxycytidine deaminases are considered the second-most prominent source of mutagenesis in sequenced tumours, next to mutations caused by ageing [11–13,24]. The association between A3 enzymes and carcinogenesis is evidenced from multi-dimensional observations. Human A3s are mainly studied using primary or immortalized cells of human or non-human origin, and the results are often cell line-specific. Also, biochemical and bioinformatic studies have provided information about the role of the A3 enzymes in the cancer field. One important observation regarding the studies of these enzymes in cancer was that the *in vivo* evaluation using a complex mammalian system was lacking in the field. Previously, no authentic animal model for the studies of individual A3 genes and proteins was reported because rodents have a single A3 gene, while humans have seven. However, the role of the transgenic expression of human A3 enzymes in two models for tumourigenesis in mice was reported recently [127], providing some *in vivo* validation to the current knowledge. In developing these two transgenic murine tumour models, it was demonstrated *in vivo* that the human A3A enzyme catalyses mutagenesis and promotes tumourigenesis in colorectal and hepatocellular carcinoma [127].

The ‘off-target’ mutations in the host genome produced by members of the A3 family, that are the basis of its relationship to cancer, has been associated with cancer development, progression, metastasis and drug resistance [31]. Most cancer cells and tumours show overexpression (20- to 60-fold) of A3B, A3A or A3H Hap I mRNA [3,5,11,12,128,129]. The efforts in characterizing the potential mutagenic activity of APOBECs have focused primarily on APOBEC mRNA expression levels, but sometimes the reported enrichment of the mutagenesis associated with a particular A3 enzyme (e.g. A3A) does not correlate with mRNA levels in tumours from the TCGA database [4] raising the possibility that expression levels alone do not determine A3 activity [29]. A key factor limiting comparisons between mutational burdens and mRNA abundance are potential differences in APOBEC expression levels at the time of mutagenesis and at the time of RNA sampling. Mutations captured in cancer genomes could have been generated by APOBEC deaminases over the lifetime of a cell lineage, whereas mRNA captures expression at the single time point of sample acquisition and not necessarily at the time of active mutagenesis [130]. Also, there is a possibility that signals of APOBEC expression in tumours originate from infiltrating immune cells with naturally higher APOBEC levels [131]. This review summarizes specific information on the leading A3s candidates (A3A, A3B and A3H Hap I) causing mutation signatures in different human cancers [3–5,13,24,132–135] taking into account both original ideas that led to the identification of APOBEC activity in cancers and the current views on A3 expression levels and levels of mutagenesis.

### 5.1. Breast cancer

BRCA is a leading cancer burden in females and the primary cause of cancer-associated deaths among women worldwide [136]. The probability of developing BRCA is modulated by the interaction of many factors such as lifestyle, environmental and genetic factors. Mutations are thought to be the key drivers of recurrence, metastasis and therapeutic resistance of cancer. The studies on the molecular origins of mutations in BRCA have implicated several mechanisms, including both spontaneous and enzyme catalysed deamination of DNA cytidine [3,9,11–13]. The former process correlates with ageing and is mostly due to hydrolytic conversion of 5-methyl cytosine (mC) bases within 5′ NmCG (N = A, C, G, or T) motifs into thymines, which escape BER and are converted into C-to-T transition mutations by DNA replication. The latter process is attributable to ssDNA deamination catalysed by one or more members of the A3 family of enzymes, characterized by C-to-T transitions and C-to-G transversions in 5′HTCW motifs (H = A, C, or T and W = A or T). The extensive resources generated by the TCGA database enabled a deep analysis of somatic mutagenesis in breast tumours [137]. Based on the patterns of mutations, at least 12 somatic mutational signatures have been annotated in TCGA breast tumours [138], two of these have been attributed to the activity of APOBEC family of proteins [11,12]. In particular, A3A and A3B have been considered the main mutagenic enzymes that generate APOBEC-signature mutations in breast and other tumour types because overexpression of these enzymes triggers DNA damage responses and inflicts chromosomal mutations in hallmark trinucleotide contexts [3,4,9,134,139]. When introducing A3A and A3B into yeast, genome-wide mutation patterns of A3A- and A3B-mediated deamination show strong similarity to mutation signatures found in BRCA, which strengthens the proposed role of A3A and A3B in BRCA hypermutation [118]. The analysis of the APOBEC-signature mutation load in cancer exons showed that it is statistically correlated with A3A and A3B transcript abundance [24]. Although A3B mRNA abundance tends to be greater than that of A3A in BRCA cancer samples, A3A is a more potent inducer of DNA damage [24]. Due to the nonlinear relationship between mRNA abundance and activity levels and different studies relying on often one or the other as a measure of A3 activity, the relative contributions of A3A and A3B to mutagenesis in BRCA cancer have been extensively debated in the literature. Collectively, the data point to both A3A and A3B being involved in BRCA, but with distinct mechanisms and effects.

**Figure 3.**
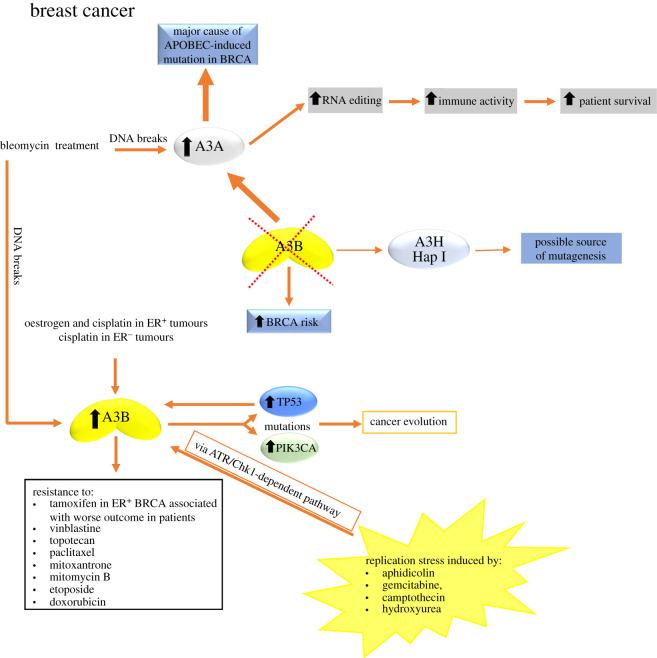
Causes and effects of A3 upregulation in BRCA. Causes of A3B upregulation could include the action of hormones (oestrogen), DNA-damaging drugs (cisplatin, bleomycin) and replication stress. The induction of A3B was found to be stronger in the presence of mutant TP53 compared to wild-type TP53. A3B upregulation contributes to increase mutations in TP53 and PIK3CA genes driving to cancer evolution and A3B upregulation is also associated with worse outcomes in cancer patients as well as with resistance to many anti-cancer drugs. In the absence of A3B, there is an increased risk to develop BRCA. A3A has emerged as a major cause of APOBEC-induced mutations in BRCA. Also, in the absence of A3B, A3H Hap I could be a source of mutagenesis. Bleomycin treatment can also upregulate A3A, although the effect is more robust for A3B. The upregulation of A3A is also associated with an increased RNA-editing activity that positively correlated with patient survival. For more details, see §5.1.

Multiple lines of evidence suggested that A3B, the only constitutively nuclear ssDNA deaminase [91], was the primary source of the mutations found in BRCA [3,11–13]. The reasoning behind these suggestions included the following observations: A3B is overexpressed in greater than 50% of breast tumours; in more than 75% of BRCA cell lines, and it was the only detectable DNA deaminase activity in BRCA cell extracts [3,13]; increased A3B levels correspond positively with overall cytosine mutational loads [3] and A3B expression associates with worse clinical outcomes of hormone therapy resistance in BRCA [140,141]. The analysis of cell line and tumour datasets showing that A3B gene expression is upregulated in malignant versus normal tissues and epithelial cell lines have shown correlations between A3B expression and the presence of certain somatic mutations, particularly in TP53 [3,142] and phosphatidylinositol-4,5-bisphosphate 3-kinase, catalytic subunit alpha (PIK3CA) (figure 3) [143]. These observations form the basis for a model where A3B expression contributes to the accumulation of somatic alterations during the process of carcinogenesis and subsequent evolution, and it has been suggested that inhibition of this activity could represent a strategy for cancer prevention or an adjuvant to other therapies [3,144]. The report that A3B expression is associated with adverse outcomes in oestrogen receptor-positive (ER^+^) BRCA would be consistent with this hypothesis (figure 3) [141] as well as the analysis of 30 human cell lines, including BRCA cell lines, from which expression levels of the A3B gene were associated with resistance to anti-cancer drugs such as vinblastine, topotecan, paclitaxel, mitoxantrone, mitomycin C, etoposide and doxorubicin (figure 3) [145].

High A3B expression in ER^+^ BRCA showed short progression-free time with tamoxifen treatment and suppression of endogenous levels of A3B enhanced tamoxifen benefit [140]. The mitogenic effect of oestrogen was supported by observations of increased cellular proliferation induced by A3B expression in BRCA cells [146]. Recently, Udquim *et al*. [147] provided some elements on the understanding of the aetiology and clinical outcomes of BRCA supporting the mitogenic hypothesis of oestrogen action independent of somatic mutagenesis (mutagenic hypothesis). Their analysis in BRCA cell lines and TCGA breast tumours suggest that A3B expression is induced by oestradiol in an ER-dependent way. They proposed that A3B expression is unlikely to account for APOBEC-mediated mutagenesis in breast tumours but might contribute to cancer development based on the possible mitogenic effect of A3B, a deamination-independent effect. The promotion of cancer progression by oestrogen-induced A3B expression affecting the tumour microenvironment in ER^+^ cells would be consistent with the proposed mitogenic effect of oestrogen [148] that is also supported by observations of increased cellular proliferation induced by A3B expression in BRCA cells [146]. The expression of A3A in the BRCA cell lines evaluated was not detectable, suggesting that A3B is an oestrogen-responsive gene and A3A is not. The expression of A3B was also induced by a DNA-damaging chemotherapy drug, cisplatin, regardless of the ER status (figure 3). Accordingly, treatment with cisplatin in women with high levels of endogenous oestrogen or receiving hormone replacement therapy may result in a further increase of A3B expression (figure 3). However, in women with oestrogen receptor-negative (ER^−^) breast tumours, endogenous oestrogen or hormone replacement therapy may not affect A3B expression, even during cisplatin therapy. p53 controls the A3B expression [149,150] and since ER^−^ tumours are enriched for inactivating p53 mutations [137,151], this could contribute to elevated levels of A3B expression in this type of tumour that is significantly higher compared with ER^+^ breast tumours. The study of Udquim *et al*. [147] has not addressed the direct causal connection between exposure to endogenous and exogenous oestrogen, A3B expression in human samples and BRCA risk. To address these relationships, it would be necessary to conduct epidemiological studies with large cohorts of BRCA patients with tumours of different subtypes taking into consideration all relevant covariates such as environmental exposures, germline and somatic variants, and clinical outcomes.

An interesting concept is that DNA damage/replication stress can induce expression of A3 genes. Expression of A3s in BRCA cell lines is inducible in response to various environmental exposures (figure 3) [95,134,147], as addressed in the study of Udquim *et al*. [147] demonstrating the A3B overexpression in response to cisplatin treatment. In addition, the issue of DNA damage/replication stress was addressed by Middlebrooks *et al*. [134] by treating three BRCA cell lines (MCF-7, MDA-MB-231 and T-47D) with bleomycin, a DNA-damaging drug known to induce DNA breaks. The study found that both A3A and A3B were induced in most cell lines, with the effect being more robust for A3B (figure 3). The induction of A3B was found to be stronger in cell lines harbouring mutant TP53 than in those with wild-type TP53 (figure 3) [134]. Following the same principle, Kanu *et al*. [95] demonstrated that chemical and cytotoxic induction of replication stress, through aphidicolin, gemcitabine, camptothecin or hydroxyurea exposure activates transcription of A3B via an ATR/Chk1-dependent pathway *in vitro* (figure 3). The role of DNA replication stress in mediating genomic instability could link the high level of somatic copy number aberrations and single nucleotide diversity caused by A3 activity that are both observed in human epidermal growth factor receptor 2 (HER2)^+^ tumours.

Within breast carcinomas, the HER2^+^ subtype has been shown to display evidence of A3-mediated mutagenesis and is associated with high levels of somatic copy number aberrations [12]. This subtype of breast tumour displays evidence of elevated levels of replication stress-associated DNA damage *in vivo*. The oncogenic signalling, cytotoxic drugs and genetic modulators of replication stress are all able to modulate A3 activity, although it has not been explored if there is a mechanistic connection between the underlying causes of chromosomal copy number aberrations and the generation of A3 mutagenesis in HER2^+^ BRCA [95]. These findings implicate the ability of therapeutics that either attenuate oncogenic signalling or exacerbate DNA replication stress to alter cancer's mutagenic landscape and evolutionary potential by multiple mechanisms.

Despite all aforementioned findings, the importance of A3B in cancer has been questioned with the observation that APOBEC-signature mutations are still clearly evident in A3B-null breast tumours (figure 3) [152]. A 29.5 kb deletion that removes the entire A3B coding sequence and fuses the 3′ untranslated regions (UTR) of A3A and A3B forms a hybrid gene that is predicted to produce a transcript which is predominantly constituted of A3A sequence but replaces the A3A 3′UTR with the A3B 3′ UTR and encodes a protein that has an identical amino acid sequence to A3A [153]. This polymorphism occurs at different frequencies in different populations around the world. The deletion allele has a frequency of approximately 8% in European populations [153,154], 37% in East Asians and 93% in Oceania [153]. Some studies showed that the A3B deletion increases the risk of BRCA [155,156] and increases tumour mutational burden [152]. A genome-wide association study in the Chinese population demonstrated the A3B deletion is associated with BRCA (odds ratio (OR) 1.3 one-copy, 1.8 two-copy deletion, *p* = 2.0 × 10^−24^) [155], which was replicated in a European population (OR 1.2 one-copy, 2.3 two-copy deletion, *p* = 0.005) [156]. However, including familial BRCA for the first time, a later study showed a lack of association of the A3B deletion with BRCA risk, which was independently validated in three European cohorts (in total: 2972 cases and 3682 controls) [157]. This study provided direct evidence for the generation of the transcriptionally active hybrid gene A3A/A3B from the allele with the A3B deletion and confirmed the suggested structure of A3A/A3B transcript, which enabled A3A, A3B and A3A/A3B expression levels to be distinguished. The knowledge of the exact structure of the hybrid transcript is vital for the design of comprehensive tests for analysis of the influence of the A3B deletion genotype on the expression of A3B, A3A and the A3A/A3B hybrid gene. A recent study showed that the germline A3B deletion influenced the APOBEC mutational signature, neoantigen loads and relative immune cell compositions in BRCA [36]. This study [36] and recent studies demonstrating that APOBEC plays an important role in promoting programmed cell death protein-1 (PD-1) expression [133,146,158,159], as well as immune activation in multiple cancer types, highlight the importance of the APOBEC genes in immunogenicity and cancer immunotherapy.

As the homozygous carriers of the A3B deletion allele are predicted not to make any A3B protein, other APOBEC enzymes must contribute to APOBEC-signature mutations during tumour development. In this regard, one study of APOBEC-induced mutations from A3B deleted BRCA tumours revealed that the only tumours displaying the APOBEC mutation signature also contained the nuclear A3H Hap I, providing correlative evidence that this protein may be the additional source of mutagenesis (figure 3) [5]. As well as for A3H Hap I [5], some evidence has implicated A3A in A3B-null BRCA (figure 3). The A3A/A3B hybrid mRNA can be expressed in the A3B-null BRCA cell line SKBR3 and this finding has suggested that A3A can contribute to a mutator phenotype in cancer (figure 3) [4,160]. The initial studies regarding A3A were done in cell lines, yeast cells or by retroactively analysing cancer genome databases for mutations without specifically examining the A3 expression patterns in A3B-null tumour cells [4,118,152,160]. Another study found the A3A mutational footprints in tumours, but no corresponding A3A expression and suggested that A3A is upregulated early, but later inactivated, perhaps due to being the most active deaminase that could cause cell death through its activity over time [4,99,161]. In addition, A3A and A3B can be differentiated by their different preferred tetranucleotide motifs, 5′YTCA and 5′RTCA (Y = T or C and R = G or A), respectively, when inducing mutations in a yeast model system [4]. However, over-representation of mutations in the 5'YTCA motif predominates in a variety of cancers [4] as well as among mutations actively acquired in BRCA cell lines [130], suggesting A3A may likewise contribute to cancer mutagenesis. A3A was often reported to have undetectable expression in BRCA lines [3,5], but more recently, some evidence indicates that A3A may be a major cause of APOBEC-induced mutation in BRCA and account for the majority of cytidine deaminase activity in extracts from multiple BRCA cell lines, despite higher A3B expression [24]. Consistent with previous reports [3,12,13,162], this study found that A3B was expressed at high levels compared to the other A3s. However, this high A3B expression level existed in both APOBEC-mutagenized and non-APOBEC-mutagenized cell lines [24], indicating that elevated A3B mRNA levels may not be directly responsible for APOBEC-induced mutagenesis as previously thought [3,12,13]. By contrast, a median 13.1-fold higher A3A mRNA expression level was observed in the APOBEC-mutated BRCA lines compared to non-APOBEC-mutated lines and the overall abundance of APOBEC-induced mutations linearly correlated with A3A expression [24]. Also, they demonstrated that A3A is the primary source of cytidine deamination activity in A3B-null AU565 and SKBR3 cell lines. A3A is more active biochemically than the next most potent somatic mutators A3B and A3H Hap I [24,79,119,163,164] and RNA binding is known to inhibit the activity of A3B and partially inhibit the activity of A3H, but not A3A [24,84,86], suggesting that A3A may be a better candidate than A3H Hap I in causing the APOBEC mutation signature in A3B null BRCA [24]. In the presence of cellular RNA, A3A contributes significantly to cytidine deaminase activity, even from extracts of BRCA cell lines with elevated A3B expression and containing A3H Hap I, indicating that in some cellular contexts, A3A is the dominant active APOBEC present in the cell. The mechanism for relieving RNA inhibition of A3B or A3H Hap I in cells is currently unknown and the A3H Hap I deaminase activity in A3B-null cells, AU565 or SKBR3, (that have at least one A3H Hap I allele) was not detected [24].

In addition to mutagenesis linked to deamination of ssDNA, A3A as well as A3B have been reported to be involved in RNA editing [19,165]. Like its preference for DNA stem-loops, A3A also recognizes RNA stem-loops in a sequence-specific manner [19]. Since A3A is much more catalytically active than A3B, but it is typically expressed at lower levels than A3B in tumours, both A3A protein and A3A mRNA are difficult to quantify. This makes it particularly challenging to predict the levels of currently ongoing A3A activity in tumours. Recently, Jalili *et al*. [166] developed a strategy using hotspot APOBEC-signature mutations in RNA stem-loops identified from A3A-positive tumours and droplet digital PCR to quantify the ongoing activity of A3A in tumours. They found that A3A expression and A3A-mediated DNA mutagenesis in tumours, but not those of A3B, correlate with APOBEC-signature mutations in RNA stem-loops. Interestingly, those RNA mutations are not present in their DNA templates, suggesting that they are directly generated by A3A. Because of the labile, transient nature of RNA, they concluded that the RNA-editing activity of A3A accurately reflects the currently ongoing activity of A3A and that the RNA mutation-based A3A assay is superior to A3A protein- and mRNA-based assays in predicting the currently ongoing A3A activity on DNA. Finally, they show that the RNA mutation-based A3A assay can be applied to clinical samples from cancer patients, providing a new opportunity to investigate the role of A3A in tumour evolution and to target A3A-induced vulnerabilities in cancer therapy [166].

In line with this RNA-editing activity of the A3 enzymes, a recent bioinformatic study identified that A3-mediated RNA editing occurs in breast tumours and is positively associated with elevated immune activity and improved survival (figure 3) [22]. Interestingly, the RNA-editing scores had the best correlation with A3A gene expression [22]. The findings of this study imply that A3 enzymes are relevant in BRCA not only because of their DNA mutagenicity but also their RNA-editing activity, and they highlight the pertinence of finer dissection of such editing in further studies. While they could detect C-to-U RNA-editing events in the tumours, the cellular origin of such events remains unclear. Overrepresentation of edited sites among immune-related genes, enriched expression of such genes in editing-high tumours, and the fact that A3 gene expression is much higher among immune compared to epithelial cells suggests that the editing occurs in the tumour microenvironment. Further investigations implementing methods such as single-cell sequencing and isolation of sub-populations of cells from tumours are needed to definitively know if the editing occurs in cancerous epithelial or immune cells of breast tumours. The biological consequences of the editing events on cancer development, progression and immune response also remain unknown.

Based on the literature, the existing data have led to an unclear understanding as to the relative contributions of individual APOBECs to mutagenesis in BRCA. A side-by-side comparison among these studies is difficult because they were not done using the same cell lines or under the same conditions. Also, it is important to elucidate which of the enzymes A3A, A3B and A3H Hap I are involved earlier in promoting cell transformation or later in promoting BRCA progression. In this regard, mechanistic studies to determine if those enzymes can deaminate the ssDNA during replication, transcription and/or DNA repair will allow us to understand better their role in BRCA.

### 5.2. Lung cancer

Lung cancer is the most common cancer in men and the third most commonly occurring cancer in women, with 2 million new cases in 2018 [167]. There are two main types: small cell lung cancer (SCLC) and non-SCLC (NSCLC). It is estimated that about 80–85% of lung cancers are NSCLC, and about 10–15% are SCLC. Also, there are three main subtypes of NSCLC: adenocarcinoma, squamous cell carcinoma and large-cell carcinoma [168]. In addition to smoking, one major cause of the heavy mutation load of NSCLC [169], the expression of APOBEC family members, especially A3B, was reported as a key source of mutations specifically in two subtypes of NSCLC: adenocarcinoma and squamous cell carcinoma [13].

**Figure 4.**
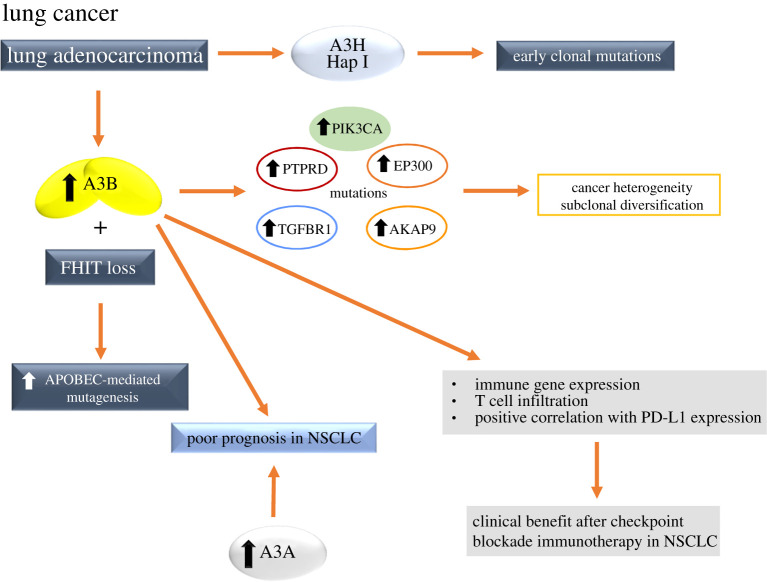
Causes and effects of A3 upregulation in lung cancer. Mutations within an A3B context were found in driver genes such as PTPRD, PIK3CA, EP300, TGFBR1 and AKAP9, fuelling cancer heterogeneity and subclonal diversification. In lung adenocarcinoma, A3H Hap I expression was associated with early clonal mutations and increased A3B expression and the loss of FHIT protein expression was associated with higher levels of APOBEC-mediated mutagenesis. The overexpression of both A3B and A3A is associated with poor clinical outcomes in NSCLC patients, but A3B overexpression also predicts clinical benefit after checkpoint blockade immunotherapy in patients. For more details, see §5.2.

A3 mutational signatures may occur at different stages in different types of cancer, contributing to later subclones in lung adenocarcinoma as the tumours evolve [170]. In an analysis of intratumour heterogeneity in early-stage NSCLC, multiregion sampling allowed the timing of mutational processes during tumour evolution to be deciphered, establishing the temporal dynamics of APOBEC mutational processes [170,171]. The enrichment of the APOBEC signature was seen in the branches of tumour evolutionary trees relative to the early clonal truncal mutations, particularly prominent in adenocarcinomas of the lung [170,171]. According to this endogenous mutational process driving subclonal expansions, mutations within an A3B context were found in driver genes such as PTPRD, PIK3CA, EP300, TGFBR1 and AKAP9 (figure 4) [170]. Also, there was evidence for spatial heterogeneity in APOBEC activity; in one adeno squamous tumour, the APOBEC signature was found enriched in the adenocarcinoma branch, harbouring driver mutations in PTPRD and TGFBR1 within an APOBEC context, but not the squamous carcinoma branch [170]. Notably, in lung adenocarcinoma and lung squamous cell carcinoma, over 85% of subclonal mutations in PIK3CA occurred in an APOBEC context [172]. Most of these subclonal mutations were found in the PIK3CA helical domain (E545 K) that have been previously linked to APOBEC-mediated mutagenesis in cervical and head/neck tumours [143]. These data highlight that both the genome instability processes and the APOBEC mutagenic process can be spatially and temporally heterogeneous during the disease course. The importance of APOBEC later in tumour evolution is highlighted by the observation that this mutational process in lung adenocarcinoma and squamous carcinoma was found to be the major source of subclonal cancer gene mutations (figure 4) [172] relative to clonal driver gene mutations, suggesting APOBEC is a mutagenic source, fuelling cancer heterogeneity and subclonal diversification. In addition to A3B, a bioinformatics approach significantly associated the cytidine deaminase A3H Hap I with clonal APOBEC-signature mutations in lung adenocarcinoma (figure 4) [5]. Later, a computational study supported this idea and identified the association of SNP rs139298, that is correlated with lung cancer and creates a K121E mutation in A3H Hap I [173]. As a follow-up to this study, the effect of the K121E mutation was assessed. First, the ability of A3H Hap I to induce DNA damage in lung cells was for the first time directly demonstrated by observing A3H Hap I-induced *γ*H2AX foci [174]. However, the K121E mutation was shown to destabilize A3H Hap I in cells and supported the conclusion that the loss of A3H Hap I activity through the K121E variant may benefit the cancer and be detrimental to the host, suggesting that A3H Hap I deamination activity can induce tumour cell death or immune recognition [174]. These data emphasize that it is important to use additional genetic or clinical data for determining the beneficial or detrimental effects of A3-induced mutations.

APOBEC-mediated mutagenesis may also be increased in the case of a reduced expression or the loss of protein activity of the tumour suppressor fragile histidine triad protein (FHIT), and higher levels of APOBEC-mediated mutagenesis were observed from TCGA lung adenocarcinoma tumours that had both increased A3B expression and the loss of FHIT protein expression (figure 4) [134,175,176]. FHIT is frequently lost very early during tumour development, causing replication stress due to deoxythymidine triphosphate depletion [177], thus the mutagenic potential of A3B may be unleashed in the absence of FHIT [176]. When genomic sequences from lung adenocarcinomas were stratified by A3B and FHIT expression, those with high A3B and FHIT loss showed significantly higher A3 signature mutation loads than high A3B expressers with normal FHIT levels [176].

Elevated A3B expression was reported to correlate with poor prognosis in lung cancers and other types of cancers, pointing to A3B as the key mutation driver in human cancers [34]. Without including A3-induced mutations, an analysis of chromosomal instability (CIN) quartiles in NSCLC has revealed that intermediate thresholds of CIN appear to exist and correlate with the poorest clinical outcomes, in contrast with excessive or minimal CIN in cases where the outcomes are better [178,179]. Also, evidence of preferential benefit from therapeutic approaches has emerged in patients with tumours with the highest mutational load [180]. This suggests that the evolutionary trade-off for increased fitness brought about by an increased mutation rate is the risk of tumour neo-antigenic presentation and immune control (figure 4) [181,182]. A3 enzymes are an important part of this balance, the effects of which appear to be specific to the tumour [34,180].

A3B-related mutational processes fuelling cancer heterogeneity and treatment resistance remains a challenge for NSCLC treatment. Although A3B upregulation is associated with poor NSCLC prognosis [34], targeting A3B in this cancer is still a big challenge. It was proposed that cancer progression would be suppressed through A3B inhibition, but there are no available drugs that can inhibit A3B expression or function. NSCLC is commonly treated by radiation, surgery and chemical therapy, but the approval of the antibodies targeting immune checkpoints PD-1 and ligand (PD-L1) had a big impact for the immunotherapy of this and other cancers. It was demonstrated in advanced NSCLC, in patients treated with an antibody targeting PD-1, response rates of 17–21% with some responses being remarkably durable [183]. Although clinical studies have shown promise for targeting PD-1, PD-L1 signalling in NSCLC, the factors that predict which patients will be responsive to checkpoint blockade are not fully understood. Based on existing publications, the predictive markers for immune checkpoint inhibitor therapy include: PD-L1 expression [184,185], tumour mutational load [181,182], DNA mismatch repair deficiency [186] and CD8^+^ T-cell activity [187,188]. A recent study was focused on the correlation of A3B expression with immune gene expression and immunotherapy response in NSCLC [133]. Although A3H Hap I has been implicated in lung cancer [5,173,174], this study stated that similar to A3B, overexpression of A3A, but not A3H, predicts poor NSCLC prognosis (figure 4) [133]. Thus, A3 family members can play distinct and overlapping functions in NSCLC. Through combined cancer genomic mutation analysis and gene expression analysis, A3B upregulation is significantly associated with immune gene expression, and A3B expression positively correlates with known immunotherapy response biomarkers, including PD-L1 expression and T-cell infiltration in NSCLC (figure 4) [133]. The APOBEC mutational signature is specifically enriched in patients with durable clinical benefit after immunotherapy and APOBEC mutation count can be better than total mutation count in predicting immunotherapy responses. This study implicates A3B and APOBEC mutational signatures as novel predictive biomarkers for checkpoint blockade immunotherapy response in NSCLC and suggests immunotherapy as a novel treatment option for A3B overexpressing NSCLC [133].

### 5.3. Bladder cancer

With almost 550 000 new cases in 2018, BLCA is considered the sixth most commonly occurring cancer in men and the 17th most commonly occurring cancer in women [167]. The risk factors for BLCA include smoking, physical inactivity, unhealthy nutrition, schistosomiasis, occupational exposure to aromatic amines, hair dye and contaminants in drinking water [189]. Urothelial carcinoma is the most common type of BLCA while squamous cell carcinoma, adenocarcinoma, small cell carcinoma and sarcoma are the less common types. BLCA is also described as non-muscle-invasive or muscle-invasive (MIBC), depending on whether it has grown into or through the muscle of the bladder wall. About 75% of patients have non-muscle-invasive BLCA and 25% have MIBC or metastatic disease. Approximately 50% of non-muscle-invasive BLCA are low grade, whereas most MIBC or metastatic tumours are high grade [190].

**Figure 5.**
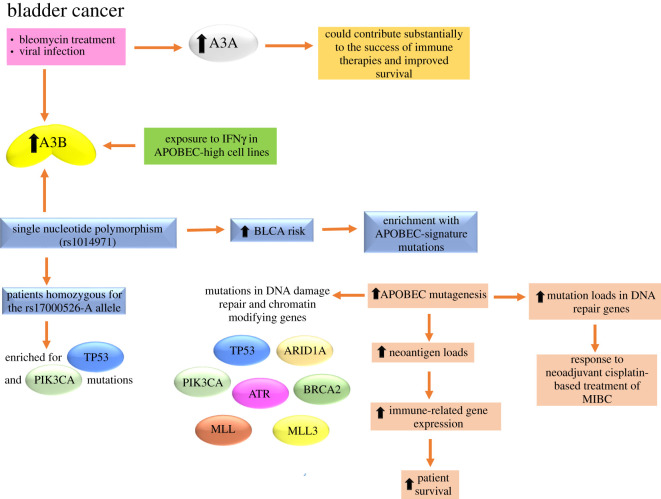
Causes and effects of A3s upregulation in BLCA. The expression of both A3A and A3B can be induced by bleomycin, a DNA-damaging agent and by an RNA virus that induces an IFN response. Also, the direct exposure to IFN*γ* in APOBEC-high cell lines increases the A3B expression. Hypermutation in BLCA (mainly by A3A) could contribute substantially to the success of immune therapies and improved survival. The single nucleotide polymorphism, rs1014971, was associated with BLCA risk, increased A3B expression and enrichment with APOBEC-signature mutations in bladder tumours. The tumours from patients homozygous for the rs17000526-A allele were enriched for TP53 and PIK3CA mutations. Increased APOBEC mutagenesis in BLCA patients have a positive association with neoantigen loads, the relative abundances of immune-related genes and the improved survival. Also, increased mutation loads, especially in DNA repair genes, were associated with a response to neoadjuvant cisplatin-based treatment of MIBC. Tumours enriched for APOBEC mutagenesis had better survival and were more likely to have mutations in both DNA damage repair and chromatin-modifying genes such as TP53, PIK3CA, ATR, BRCA2, MLL, MLL3 and ARID1A. For more details, see §5.3.

APOBEC mutagenesis is the predominant mutational pattern in BLCA [11–13]. About 80% of bladder tumours in the TCGA have an APOBEC mutation signature that is also frequently found in BRCA, lung, head and neck, and cervical cancers [3,11–13,134,191]. Although several studies have been focused on the linkage of A3B expression with mutagenesis [3,13,101], its expression alone does not fully explain the APOBEC mutational signature, and A3A can play a significant role as recently demonstrated for BRCA [4,24] and also for BLCA [36,134,135]. Middlebrooks *et al*. in 2016 [134] demonstrated that expression of both A3A and A3B can be induced in BLCA cell lines (HT-1376, HTB-9 and RT-4) that represent some of the major clinical subtypes of bladder tumours by bleomycin, a DNA-damaging agent and by an RNA virus that induces an interferon (IFN) response [134] (figure 5). Both A3A and A3B were induced in all cell lines by bleomycin, but the effect was more robust for A3B. By contrast, A3A expression was uniformly induced four- to 167-fold by viral infection with in BLCA cell lines. This range of induction suggests additional cell-type-specific factors that may affect sensitivity to different environmental exposures [134]. The analysis of TCGA BLCA patient datasets revealed that a single nucleotide polymorphism, rs1014971, but not the germline A3A/A3B deletion [36], was associated with BLCA risk, increased A3B expression and enrichment with APOBEC-signature mutations in bladder tumours (figure 5). Also, this group demonstrated that TCGA BLCA patients with increased APOBEC mutagenesis had significantly improved survival, and that the tumours from patients homozygous for the rs17000526-A allele were enriched for TP53 and PIK3CA mutations [134] (figure 5). The more efficient immune surveillance due to neoantigens and synthetic lethality of tumour cells could contribute to improved survival for patients with higher APOBEC mutagenesis. In line with this observation, other groups reported a significant positive association between APOBEC mutational signature and neoantigen loads in BLCA [36,192] that also has an association with the relative abundances of immune-related gene expression [36,135] (figure 5). When evaluating the effects of all A3 isoforms on survival, the effect of A3B expression was comparable to that of rs17000526 and similar in treated and untreated patients, while the effect of A3A expression was much stronger in treated compared to untreated patients suggesting that mutagenesis caused by A3B may represent a genetically regulated mechanism contributing to cancer initiation, while mutagenesis caused by A3A may represent events occurring in tumours and influenced by the tumour-specific environment, including treatment [134]. Increased mutation loads, especially in DNA repair genes, were also associated with a response to neoadjuvant cisplatin-based treatment of MIBC (figure 5). This was attributed to the inability of cancer cells to recover after treatment-induced DNA damage [134].

Robertson *et al*. in 2017 [192] highlighted essential findings from the complete cohort of 412 MIBC samples characterized by multiple TCGA analytical platforms. They confirmed that MIBCs show high overall mutation rates similar to those of melanoma and NSCLC, and these high rates are principally associated with mutation signatures from APOBEC enzymes [134]. Most BLCA mutations are clonal, suggesting that APOBEC's mutagenic activity occurs early in BLCA development [134,172,192]. For instance, mutations in specific cancer genes as TP53 and ARID1A show a tendency to be clonal, but focusing on subclonal mutations in known cancer driver genes, in APOBEC-associated BLCA more than 45% of subclonal mutations in driver genes occurred in an APOBEC context [172]. Curiously, in BLCA, it appears that one of the two A3 mutation signatures defined by Alexandrov and co-workers (signature 13) is enriched early, while the other (signature 2) becomes enriched in subclones [11,172]. A better understanding of the origin and regulation of APOBEC expression and activity in normal bladder could lead to preventive strategies that target APOBEC as a key mutagenic source in BLCA. Robertson *et al*. in 2017 [192] also corroborated a positive correlation between the improved survival of subjects with higher mutational APOBEC burden and higher neoantigen load as described by Middlebrooks *et al*. [134] and proposed that this is due to a natural host immune reaction to the high mutation burden, curbing further tumour growth and metastasis [192]. Also, chromatin modifier gene mutations are common in BLCA and open potential therapeutic opportunities through rebalancing acetylation and deacetylation, and through other chromatin modifications. Integrating RNA subtype classification, pathway information, epithelial–mesenchymal transition and low carcinoma-*in situ* signatures, and immune infiltrate analyses led to proposal of a model of mRNA-based expression subtypes that may be associated with unique responses to therapies that can be prospectively tested in clinical trials [192]. Neoadjuvant cisplatin-based chemotherapy is the current standard of care in cisplatin-eligible patients without risk stratification. However, as not all patients derive benefit from chemotherapy, subtype-specific personalized therapies could help to optimize global patient outcome, while preventing unnecessary toxicity to non-responders. Also, the results obtained by this group suggested that mRNA subtype classification may be possible with a reduced gene set, enabling validation in independent cohorts and informing clinical trial designs that test new personalized therapies [192].

Recently, several groups investigated the APOBEC mutational signature in the TCGA, Beijing Genomics Institute and Cancer Cell Line Encyclopedia BLCA datasets and its relationship with specific mutations, molecular subtype, gene expression and survival. The results obtained by Glaser *et al*. in 2018 [135] were in good agreement with the results obtained by Middlebrooks *et al*. in 2016 [134] and also with those obtained by Robertson *et al*. in 2017 [192]. They hypothesized that tumours with high levels of APOBEC-mediated mutagenesis would be enriched for mutations in DNA damage response genes and express genes related to activation of the immune system at higher levels, while tumours with low levels of APOBEC-mediated mutagenesis may have enrichments for oncogenes. They found that the expression of A3A and A3B were the only APOBEC enzymes that directly correlated with the total mutation burden in every BLCA subtype. A3A was expressed at a significantly higher level in the basal subtype than in luminal, p53-like or claudin-low subtypes, while A3B was evenly expressed across subtypes. Tumours enriched for APOBEC mutagenesis had better survival and were more likely to have mutations in both DNA damage repair and chromatin-modifying genes such as TP53, PIK3CA (primarily at E542 K and E545 K), ATR, BRCA2, MLL, MLL3 and ARID1A (figure 5). The APOBEC mutagenesis signature was associated with immune signatures and with increased expression of immune-related genes [135]. Bladder tumours not enriched for APOBEC mutagenesis were more likely to have mutations in FGFR3 and the RAS family of oncogenes (KRAS/HRAS/NRAS), which are mutually exclusive, and these patients had poor overall survival. The mutational pattern described above was also confirmed by analysis of 20 BLCA cell lines [135]. Finally, to further evaluate the association of APOBEC mutational pattern, A3B enzyme expression and the immune environment, they analysed A3B expression in two APOBEC-low cell lines (RT4 and KU-19–19) and two APOBEC-high cell lines (HT-1376 and UM-UC-3) after exposure to IFN*γ*. The expression of A3B increased after exposure to IFN*γ* in APOBEC-high cell lines (figure 5), but not in APOBEC-low cell lines suggesting that urothelial cancers with high APOBEC activity may have a feed-forward mechanism resulting in increased APOBEC expression upon immune activation [135].

As mentioned for lung cancer, a preferential benefit from therapeutic approaches has emerged in patients with tumours with a high prevalence of APOBEC mutagenesis. It has been suggested that hypermutation could enhance the effectiveness of immune stimulation therapy to treat cancer, by means of the generation of tumour-specific neoantigens that might trigger targeted destruction by the immune system. BLCA often has high levels of enrichment for APOBEC mutagenesis and A3A-like signatures [4]. The clinical observations in BLCA patients treated with available immune therapies [193,194] raise the intriguing possibility that hypermutation in BLCA (mainly by A3A) could contribute substantially to the success of immune therapies [4].

### 5.4. HPV-associated cancers

HPVs are small, non-enveloped double-stranded DNA viruses consisting of an 8 kb circular genome encased in a viral capsid [195]. There are more than 300 different genotypes, as well as thousands of variants, many of which may have been generated because of A3-induced mutagenesis [196]. These viruses have tropisms for the cells in the basal layer of either cutaneous or mucosal epithelia, in which the viral life cycle is tightly linked to and dependent upon keratinocyte differentiation [197]. At least 14 HPV types are carcinogenic, and these ‘high-risk’ (HR) types, among which HPV16 and HPV18 are the most studied, cause human cancers in the mucosal epithelia of several sites, including the cervix, vulva, vagina, penis, anus, and head and neck, especially those from the oropharynx that includes the tonsils and tongue base [198,199]. HPV16 infection is a major risk factor in cervical, head and neck squamous cell carcinoma (HNSCC) and oropharyngeal cancers. Particularly, cervical cancer has received the most attention because it ranks as the fourth leading cause of female cancer in the world with about 570 000 new cervical cancer cases diagnosed annually and 311 000 deaths [200].

A3s enzymes are all expressed, albeit at vastly different levels, in epithelial cells, which are the natural hosts of HPV infection [201]. While their cytidine deaminase activity causes C-to-T mutations during viral genome synthesis, A3s also restrict viral replication through cytidine deaminase-independent mechanisms [42]. Surprisingly, HR-HPVs have not evolved strategies to counteract restriction by A3 enzymes. The strong enrichment of the APOBEC signature in cervical cancer exomes [11–13] and the previous evidence for A3 editing of HPV genomes in plantar warts and pre-cancerous cervical lesions [202] suggest that the presence of HPV in cells might somehow induce or potentiate A3 activity, damaging the host genome and resulting in the observed enrichment of these mutational signatures in HPV-associated cancers [203].

**Figure 6.**
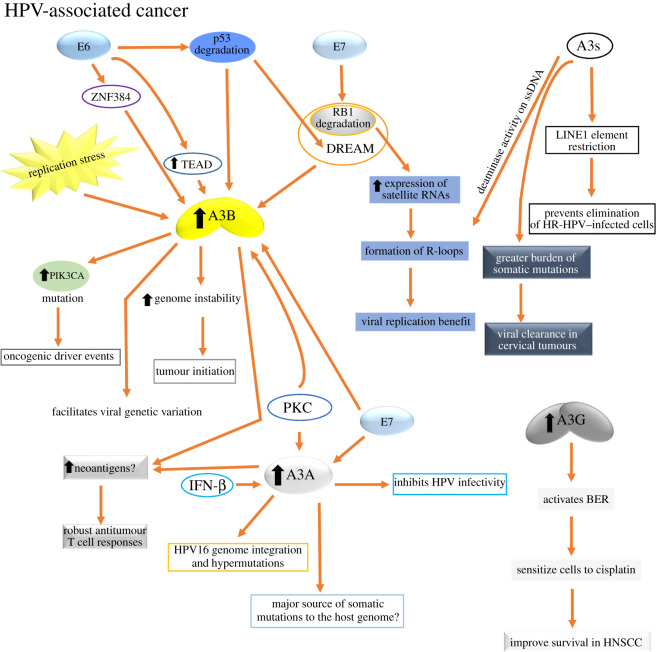
Causes and effects of A3s upregulation in HPV-associated cancer. In these types of cancers, the downregulation of pRB and p53 tumour suppressors exerted by E7 and E6 oncoproteins, respectively, seems to be the main mechanisms by which the mutagenic activity of A3B and A3A is triggered. Other causes for A3B upregulation are replication stress and PKC signalling activation, with the latter being also a cause for A3A upregulation. The upregulation of A3B may benefit the virus by facilitating viral genetic variation or by helping to transform the cells. It was proposed that A3A, rather than A3B, may be the major source of somatic mutations to the host genome in HPV-associated cancer. Abundant neoantigens in these types of cancers may be associated with the deaminase activity of upregulated A3A and A3B expression that may be beneficial for tumour immunotherapy. A3A is also involved in the inhibition of the HPV infectivity, genome integration and hypermutation. The activity of A3s against retroelements could ameliorate the loss of LINE1 silencing caused by E7 inhibition of RB1. Also, HR-HPV-mediated RB1 degradation causes high-level expression of satellite RNAs that can lead to the formation of R-loops. A3s target the ssDNA in R-loops and can thereby also activate the DNA damage response, which benefits the virus replication. Cervical infections with a greater burden of somatic HPV16 A3-induced mutations are more likely to be benign or subsequently cleared. Lastly, higher A3G expression in HNSCC activates BER, sensitizing the tumour cells to cisplatin treatment and, consequently, improving the patient survival. For more details, see §5.4.

Proteins E6 and E7 from HR-HPV types are oncogenes that are important for carcinogenesis and have some key activities such as the induction of replication stress, host DNA repair responses and downregulation of the pRB and p53 tumour suppressors [204] that may serve to trigger the mutagenic activity of A3 proteins seen in HPV-associated cancers (figure 6). Several studies indicate that NF-κB pathway activation, p53 inactivation by HPV oncoprotein E6 activation, or loss-of-function mutations in the TP53 gene and replication stress activation are responsible for transcriptional activation of APOBEC, in particular, A3B (figure 6) [95,149,205,206]. Both the E6 and E7 proteins from HPV16 can act independently to increase A3B expression in immortalized keratinocytes through this pathway; E6 via p53 degradation, with E7 likely acting through its effects on the p107 and p130 pRb family pocket proteins in the DREAM (DP1, RB-like, E2F4 and MuvB) complex [150], thus also offering a mechanistic basis for the E7-mediated A3B upregulation previously described (figure 6) [207]. The loss of p53 activity through mutations (e.g. in BRCA [3,142,146]) or HPV-16 E6/E7-mediated downregulation, causes A3B upregulation. Thus, inactivation of p53 by viral protein E6 activation or loss of function of p53 mutations can activate A3B function, increase genome instability and promote tumour initiation. Removing p53 allows HPV not only to activate A3B transcription, but possibly also allows the A3B protein to accumulate to levels that would not otherwise be tolerated in normal cells.

In addition to the role of HPV E6/E7 described above in upregulating A3B, it was recently proposed that the activity of A3s against retroelements could ameliorate the loss of LINE1 silencing caused by E7 inhibition of RB1, thus providing a potential explanation for why HPV causes A3 upregulation (figure 6) [208]. A3s are well known to restrict expression of repetitive elements, including LINEs, via predominantly deaminase-independent activity [68]. RB1 protein plays a key role in the epigenetic silencing of repetitive elements that may provide an alternative explanation as to why it may be beneficial for HR-HPVs not to counteract A3 restriction (figure 6). RB1 silences repetitive elements by associating with a unique E2F1 transcription factor complex and the degradation of RB1 by HR-HPV E7 proteins is, therefore, predicted to cause transcription of repetitive elements [209]. Translation of LINE1 results in neoantigen expression, so transcription of repetitive elements would put HR-HPV-infected cells at risk of extinction through adaptive immune responses. A3 restriction of repetitive elements may protect HR-HPV-infected cells from undergoing excessive, lethal DNA damage and genomic instability and will prevent the elimination of HR-HPV-infected cells by adaptive immune responses to neoantigen expression due to expression of repetitive elements. Therefore, HR-HPV-infected cells would gain significant advantages from this A3-dependent restriction of LINE elements. Also, HR-HPV-mediated RB1 degradation causes high-level expression of satellite RNAs that can lead to the formation of R-loops, which causes replication forks to stall [209]. A3s target the ssDNA in R-loops and can thereby also activate the DNA damage response [210], which benefits virus replication (figure 6).

Other mechanisms of A3 dysregulation have been described in the context of viral carcinogenesis. HPV16 or HPV18 induces A3B expression in cultured cells of BRCA and HNSCC, and the virus-encoded protein E6 directly binds the A3B promoter and triggers transcription. Two functional regions responsive to E6 have been identified in the promoter: a distal region (from −200 to −51), required for basal promoter activity, and a proximal region (from +1 to +45), which exerts an inhibitory effect on gene expression. Through the regulatory functions of the cellular zinc finger protein ZNF384, E6 relieves this inhibition [211] and interacts with transcriptional-enhanced associate domain (TEAD) transcription factors at the distal region [212]. E6-mediated p53 degradation, therefore, not only de-represses A3B transcription via the DREAM complex, but also results in increased levels of TEAD expression, further activating the A3B promoter (figure 6). Infection with several polyomaviruses specifically upregulated A3B expression and activity and the viral T antigen was shown to be sufficient to mediate this response [43], suggesting that A3B upregulation appears to be a conserved response to small DNA tumour viruses.

Upon inspection of the genes most frequently A3-mutated in HPV-associated cancers, the PIK3CA proto-oncogene is almost exclusively mutated at two helical domain hotpots [143]. The distribution of PIK3CA-activating mutations is different in head and neck cancers, with exclusively helical domain C-to-T transitions observed in HPV-positive tumours and a combination of helical domain and kinase domain mutations in HPV-negative tumours [143]. The HPV-positive tumours have 5′-TGA-to-TAA transitions (complementary strand 5′-TCA-to-TTA) that convert both helical domain Glu542 and Glu545 to Lys, whereas HPV-negative tumours often have a 5′-CAT-to-CGT transition mutation resulting in a kinase domain His1047 to Arg substitution. Similar helical domain biases have also been reported for PIK3CA mutations in other APOBEC-signature tumour types, implying as the common denominator the APOBEC mutagenesis and not viral infection [143,172]. This mutation distribution in the PIK3CA oncogene is significant because it implies that A3B is the predominant source of the helical domain mutations [213], although the other A3s have not been investigated. The distinctive pattern of APOBEC-signature mutations in exon 9 of the PIK3CA proto-oncogene in HPV-positive HNSCC and in other cancer types displaying the APOBEC mutational signature implicates APOBEC activity in the generation of oncogenic driver events [143,214], findings that were confirmed by analysis of TCGA HPV-positive HNSCC cohorts [215,216]. The strong link between A3B upregulation and viral infection, specifically HPV in HNSCC, cervical and perhaps some bladder tumours, suggests that other cancer types may have an unknown association with a virus or DNA-based genomic parasite. The upregulation of A3B may benefit the virus by facilitating viral genetic variation or by helping to transform the cell and thereby increase the virus's chances of spreading (figure 6). There are also likely to be non-viral mechanisms for A3B upregulation, and understanding what activates APOBECs is particularly important, as it could help in the development of methods to prevent activation. Activation of PKC signalling during differentiation of HPV-infected keratinocytes is a likely means by which at least A3A and possibly A3B could become upregulated (figure 6) during productive HPV infections, potentially triggering viral genome editing alongside viral amplification [28].

As recently described for BRCA [24], A3B mRNA levels are not correlated with A3 signature mutation burden in cancers associated with the HPV infection [143,217], but A3B expression is consistently elevated in HPV-associated cancers in comparison to both normal tissue and to HPV-independent cancers arising at equivalent anatomical sites [214]. It could be that when the mutations are occurring during development of these tumours, they are correlated with the expression of the A3 responsible, but that this relationship is lost following subsequent downregulation, possibly because of the role of A3s as transient hypermutators [129]. The preponderance of A3-induced mutations in HPV-driven cervical cancer, together with the observation that A3-induced mutations are enriched in the HPV-associated subset of HNSCC, suggest a possible off-target response to the virus [12,13,143,217]. Consistent with this, HPV16 infection upregulates by means of E7 the expression of A3A and A3B mRNA in keratinocytes, and both enzymes are upregulated in pre-invasive cervical lesions [207,213]. E7 from HR-HPV types can stabilize A3A protein by blocking its polyubiquitination by cullin-RING-based E3 ubiquitin ligase complexes; thus, HPVs appear to modulate A3 expression at multiple levels (figure 6) [218]. Also, A3A expression is associated with HPV16 genome integration and hypermutations in oropharyngeal cancers [219]. These observations and the previous studies demonstrating A3B upregulation by HR-HPV types [207,213] suggest an important role for A3B not only in HPV-associated cancer but also possibly in the viral life cycle. Periyasamy *et al*. [162] have shown that A3B associates with the ER in BRCA cell lines and co-activates ER target genes, involving deamination of promoter sites by A3B, leading to recruitment of DNA repair proteins and local chromatin remodelling. The cervical epithelium is an oestrogen-responsive tissue; indeed, HPV E6/E7-driven cervical cancer development in transgenic mice can be promoted by oestradiol infusion over several months [220]. Considering these factors, it is a possibility that A3B could also fuel cervical carcinogenesis via this non-mutagenic but nonetheless deaminase-dependent transcriptional activity.

The regulation of A3B by HPV has been the focus of attention; however, it is important to consider the roles that other A3 genes may play, both in the response to HPV infection and in HPV-associated cancer. Vartanian *et al*. [202] first reported the evidence for APOBEC editing of HPV in human cells and noted that HPV1a DNA co-transfected with A3A, A3C and A3H but not A3B displayed evidence of cytidine deamination, and while low-risk HPV genomes isolated from warts display evidence of A3 editing, several tested low-risk E6 variants did not upregulate A3B in cultured keratinocytes [213]. Taken together these findings [202,213] and the study from Warren *et al*. [207] suggesting that A3A but not A3B inhibits HPV infectivity, there were two hypotheses considered. The first hypothesis considered the possibility that the A3 response to HPV infection (mediated by A3A and/or A3C, A3H) is entirely separate from any role in host mutagenesis (mediated by A3B) during cancer development. The other hypothesis was that although A3B is induced by HPV, it is not responsible for the mutations seen in either viral or host genomes. Consistent with this hypothesis is the analysis of tumour exome data, in which there was much greater enrichment of the 5′YTCA (A3A) signature across multiple tumour types including cervical cancer [4]. That apparent preference of A3A is also supported by *in vitro* studies using purified enzyme [221,222]. Although further investigations will be necessary, these observations suggest A3A, rather than A3B, may be the major source of somatic mutations to the host genome in HPV-associated cancer. Also, A3A could contribute to the loss of HPV genomes in persistently infected cells, given that A3A is an IFN-inducible protein in keratinocytes [223], and that A3A can eliminate foreign DNA [52].

The treatment with IFN-β significantly restricts HPV infection in keratinocytes as well as represses HPV DNA replication in infected keratinocytes [207,224–227]. A3s are IFN-inducible proteins that target retroviruses and DNA viruses, so Wang *et al*. studied whether A3s are also involved in the IFN-β-mediated response against HPV infections. This study showed that IFN-β treatment upregulated A3A expression in cervical keratinocytes, and that knockdown of A3A expression reduced IFN-β-induced hypermutation of the viral E2 gene (figure 6) [223]. Using a high-yield HPV production system, it has been shown that virions packaged in cells overexpressing A3A are dramatically less infectious in keratinocytes [228]. By contrast, the expression of other A3s localized to the nucleus (A3B and A3C) had no effect on restricting viral infection [207]. HPV restriction by A3A is deaminase-dependent, as a catalytically inactive mutant A3A was unable to restrict HPV infection [207]. Further analysis of whole viral genome or RNA sequences are needed to identify critical A3A mutation targets that disrupt HPV infectivity.

Several clinical trials have shown that HPV-positive HNSCC patients have a better response to chemotherapy and radiation therapy than HPV-negative cases [229,230]. The reasons for this different behaviour can be found in the opposite genetic features which characterize these two types of tumours. Cisplatin is used for the HNSCC treatment to induce DNA adducts including interstrand cross-links (ICLs) and previous reports have shown that HPV-positive HNSCC patients respond better to cisplatin therapy. The loss of BER and mismatch repair (MMR) results in cisplatin resistance and UNG is required to initiate the BER response to cisplatin treatment and maintain drug sensitivity. Specific cytidine deaminases could play an important role in the cisplatin response by activating the BER pathway to mediate drug sensitivity. A recent study used TCGA HNSCC data to assess the association between the expression of the seven proteins in the A3 cytidine deaminase family, HPV-status and survival outcomes [231]. Higher A3G expression in HPV-positive tumours corresponds with better overall survival (figure 6). The results obtained suggest that A3s activate BER in HNSCC, mediate repair of cisplatin ICLs and thereby, sensitize cells to cisplatin which likely contributes to the improved patient responses observed in HPV-infected patients [231].

Contrary to the idea that A3-mediated somatic mutations may drive HPV-positive cancer progression, recent cancer immunology studies have shown that high levels of somatic mutations favour anti-tumour immune responses that also coincide with better prognosis after immunotherapies [232–234]. Tumour neoantigens are recognized as emerging targets for personalized cancer immunotherapies, implying that cancers with a high level of A3 mutation signatures may be beneficial for immunotherapies that induce robust anti-tumour T-cell responses specific to neoantigens generated by A3-mediated mutations. One study has revealed that tumour-infiltrating lymphocytes in cervical cancer are more reactive to neoantigens than to HPV viral epitopes [235]. This finding suggests that abundant neoantigens in HPV-positive cancers may be associated with the deaminase activity of upregulated A3A and A3B expression (figure 6). In this regard, A3-mediated mutations could be used beneficially to identify T-cell epitopes and treat HPV-positive cancer patients. Also, Boichard *et al*. [159] found that ‘kataegis’ and A3 overexpression participate in regulation of PD-L1 expression. Thus, it would be interesting to investigate if A3 mutation loads in patients correlate to better outcome following current immunotherapies targeting immune checkpoint blockades, e.g. PD-L1 that is not expressed in normal cervical tissue but is expressed in 95% of cervical intraepithelial neoplasia (grades 1 and 2) [236]. Lastly, one study analysed the viral genomes of 5328 HPV16-positive case–control samples to investigate mutational signatures and the role of human A3-induced mutations in viral clearance and cervical carcinogenesis [132]. This analysis revealed that cervical infections with a greater burden of somatic HPV16 A3-induced mutations are more likely to be benign or subsequently clear (figure 6), suggesting they may reduce persistence, and thus progression, within the host [132].

## 6. Concluding remarks

Despite the intense research completed to delineate the specific contribution of each A3 enzyme involved in cancer, this is still an area that requires further investigation. In general, the studies have been performed in yeast, cell lines and tumours using bioinformatics resources. The comparison among them has been difficult because those studies were done under different conditions or using different cell lines. In some cases, though not conclusively, we are gaining some knowledge about the relationship between A3 enzymes and cancer. However, the mechanistic studies informing us whether A3s are associated with the cell transformation or which A3s are involved in tumour progression are lacking in the field. Also, the evaluation in a complex system using animal models will be necessary because the cell culture cannot mimic the interactions that take place *in vivo*, particularly when studying enhanced immune recognition due to A3-induced neoantigens.

The leading candidates for inducing mutations in cancer are A3B, A3A and A3H Hap I. Notably, according to the current knowledge, the role of each A3 needs to be evaluated in each cancer separately because it is not obvious if one finding translates to additional cancer types. A3B, for example, could provide a mutation rate that is in some cases beneficial favouring immune responses following immunotherapy with a positive outcome for the cancer patients [133], but in other cases, it is related with poor clinical outcomes [34,140,141]. Understanding the role of A3A in cancer evolution, drug resistance or response to therapies requires further investigation due to the latest research suggesting its prominent role in BRCA [24], which was previously attributed to A3B [3,11–13] and the effect of A3A expression in BLCA which was much stronger than A3B in treated versus untreated patients and was associated with improved survival [134]. The role of the cytidine deaminase A3H Hap I in cancer has been less studied, but it was associated with BRCA and lung cancer [5,173,174]. Table 1 summarizes the beneficial and detrimental outcomes of the A3s overexpression. This table and the pathways of A3 activation (figures 3–6) that have emerged from different cancers exemplify the complexity of discerning the role of each A3 in each specific cancer. Additionally, table 2 summarizes where A3s candidates have been related to mutagenesis, prognosis, carcinogenesis or cancer progression for the cancers included in this review.

**Table 1.** Beneficial and detrimental outcomes of the A3s in cancer.

beneficial outcomes	detrimental outcomes
— A3-mediated RNA editing is positively associated with elevated immune activity and improved survival in BRCA [22] — germline A3B deletion influence APOBEC mutational signature, neoantigen loads and relative immune cell compositions in BRCA that is beneficial for cancer immunotherapy [36] — A3B and APOBEC mutational signature is specifically enriched in patients with durable clinical benefit after immunotherapy in NSCLC [133] — increased APOBEC mutagenesis in BLCA patients have a significant positive association with neoantigen loads, the relative abundances of immune-related gene expression and the improved survival [36,134,135,192] — increased mutation loads, especially in DNA repair genes, were associated with responsiveness to neoadjuvant cisplatin-based treatment of MIBC [134] — hypermutation in BLCA (mainly by A3A) could contribute substantially to the success of immune therapies [4] — higher A3G expression sensitizes cells to cisplatin which likely contributes to the improved patient responses observed in HNSCC [231] — tumour-infiltrating lymphocytes in cervical cancer are more reactive to neoantigens than to HPV viral epitopes [235], and the abundance of those neoantigens could be associated with the deaminase activity of upregulated A3A and A3B expression — cervical infections with a greater burden of somatic HPV16 A3-induced mutations are more likely to be benign or subsequently clear [132]	— A3B overexpression is associated with adverse outcomes in ER^+^ BRCA [140,141] — A3B deletion increases the risk of BRCA [155,156] and increases tumour mutational burden [152] — APOBEC is a mutagenic source, fuelling cancer heterogeneity and subclonal diversification in lung adenocarcinoma, squamous cell carcinoma, BRCA, HNSCC and BLCA [172] — A3B upregulation is associated with poor NSCLC prognosis [34] — A3H Hap I may cause early clonal mutations in lung adenocarcinoma [5] — the loss of A3H Hap I activity through the K121E variant may benefit the lung cancer and be detrimental to the host [174] — the single nucleotide polymorphism, rs1014971, was associated with BLCA risk [134] — A3A expression associates with HPV16 genome integration and hypermutations in oropharyngeal cancers [219]

**Table 2.** A3 candidates involved in mutagenesis, prognosis, carcinogenesis or cancer progression in BRCA, lung cancer, BLCA and HPV-associated cancer.

	A3s candidates for			
	BRCA	lung cancer	BLCA	HPV-associated cancer
mutagenesis	A3A and A3B [3,4,9,24,134,139], A3H Hap I [5]	A3A and A3B [4,134,175,176], A3H Hap I [5]	A3A and A3B [4,134,135]	A3A and A3B [4,28,207,213]
prognosis	A3B [140,141]	A3A and A3B [34,133]	A3A [134]	A3-induced mutations [132,134]
carcinogenesis	A3B [3,144]			A3B [220]
cancer progression	A3B [147]			

Undoubtedly, knowledge of A3 mutagenesis in cancer may yield significant diagnostic and prognostic value and could open the doors towards new therapeutic opportunities. Understanding what activates APOBECs is also of critical importance, as it could allow the development of methods to prevent their activation. Given evidence for the ‘just-right’ threshold of genome instability in cancers, increasing genomic instability in APOBEC-high tumours may also be beneficial. We are only at the beginning of research on the effects of APOBEC-mediated mutagenesis in cancer and many questions remain to be answered. In this sense, we want to leave open some questions for further consideration: (i) is overexpression of A3 enzymes the cause for cancer initiation or is it a consequence during cancer development? (ii) How and when can we avoid the off-target A3-induced mutations that results in poor clinical outcomes, and (iii) why does A3 overexpression sometimes have a clinical benefit for cancer patients? All the knowledge about the relationship between A3 enzymes and cancer will be decisive for combined therapy in cancer and will hopefully lead to refined personalized therapy in future years.

## Data accessibility

This article has no additional data.
